# The integrin beta1 modulator Tirofiban prevents adipogenesis and obesity by the overexpression of integrin-linked kinase: a pre-clinical approach in vitro and in vivo

**DOI:** 10.1186/s13578-022-00746-1

**Published:** 2022-01-28

**Authors:** S. de Frutos, M. Griera, M. Hatem-Vaquero, S. Campillo, E. Gutiérrez-Calabres, D. García-Ayuso, M. Pardo, L. Calleros, M. Rodríguez-Puyol, D. Rodríguez-Puyol

**Affiliations:** 1 Departamento de Biología de Sistemas, Unidad Fisiología, Facultad de Medicina, Universidad de Alcalá, A2 KM 33,600, Campus, 28805 Alcalá de Henares (Madrid), Spain; 2 Instituto Ramon Y Cajal de Investigación Sanitaria, Fundación Renal Iñigo Álvarez de Toledo and RedInRen from Instituto de Salud Carlos III, and NOVELREN from Comunidad de Madrid, Madrid, Spain; 3 Graphenano Medical Care S.L., Madrid, Spain; 4 Biomedical Research Foundation from Hospital Universitario Príncipe de Asturias, Alcalá de Henares, Spain; 5 Instituto de Investigación Sanitaria de Santiago, Servizo Galego de Saúde, A Coruña, Spain; 6 Nephrology Unit from Hospital Príncipe de Asturias and Department of Medicine from Universidad de Alcalá, Alcalá de Henares, Spain

**Keywords:** Tirofiban, Integrin-beta1, Integrin-linked kinase, AKT, Adipogenesis, Obesity, Hypertrophic adipocyte, Browning, White adipose tissue, Lipolysis, Inflammation

## Abstract

**Background:**

Obesity is caused by the enlargement of the white adipose tissue (WAT) depots, characterized by the hypertrophic enlargement of malfunctioning adipocytes within WAT which increases the storage of triglycerides (TG) in the lipid droplets (LD). Adipogenesis pathways as well as the expression and activity of some extracellular matrix receptors integrins are upregulated. Integrinβ1 (INTB1) is the main isoform involved in WAT remodeling during obesity and insulin resistance-related diseases. We recently described Integrin Linked Kinase (ILK), a scaffold protein recruited by INTB1, as an important mediator of WAT remodeling and insulin resistance. As the few approved drugs to fight obesity have brought long-term cardiovascular side effects and given that the consideration of INTB1 and/or ILK modulation as anti-obesogenic strategies remains unexplored, we aimed to evaluate the anti-obesogenic capacity of the clinically approved anticoagulant Tirofiban (TF), stated in preclinical studies as a cardiovascular protector.

**Methods:**

Fully differentiated adipocytes originating from C3H10T1/2 were exposed to TF and were co-treated with specific INTB1 blockers or with siRNA-based knockdown ILK expression. Lipid-specific dyes were used to determine the TG content in LD. The genetic expression pattern of ILK, pro-inflammatory cytokines (MCP1, IL6), adipogenesis (PPARγ, Leptin), thermogenesis (UCP1), proliferation (PCNA), lipid metabolism (FASN, HSL, ATGL), and metabolite transporters (FABP4, FAT, AQP7) were detected using quantitative PCR. Cytoskeletal actin polymerization was detected by confocal microscopy. Immunoblotting was performed to detect INTB1 phosphorylation at Thr788/9 and ILK activity as phosphorylation levels of protein kinase B (AKT) in Ser473 and glycogen synthase kinase 3β (GSK3β) at Ser9. TF was intraperitoneally administered once per day to wildtype and ILK knockdown mice (cKDILK) challenged with a high-fat diet (HFD) or control diet (STD) for 2 weeks. Body and WAT weight gains were compared. The expression of ILK and other markers was determined in the visceral epididymal (epi) and inguinal subcutaneous (sc) WAT.

**Results:**

TF reduced TG content and the expression of adipogenesis markers and transporters in adipocytes, while UCP-1 expression was increased and the expression of lipases, cytokines or PCNA was not affected. Mechanistically, TF rapidly increased and faded the intracellular phosphorylation of INTB1 but not AKT or GSK3β. F-actin levels were rapidly decreased, and INTB1 blockade avoided the TF effect. After 24 h, ILK expression and phosphorylation rates of AKT and GSK3β were upregulated, while ILK silencing increased TG content. INTB1 blockade and ILK silencing avoided TF effects on the TG content and the transcriptional expression of PPARγ and UCP1. In HFD-challenged mice, the systemic administration of TF for several days reduced the weight gain on WAT depots. TF reduced adipogenesis and pro-inflammatory biomarkers and increased lipolysis markers HSL and FAT in epiWAT from HFD, while increased UCP1 in scWAT. In both WATs, TF upregulated ILK expression and activity, while no changes were observed in other tissues. In HFD-fed cKDILK, the blunted ILK in epiWAT worsened weight gain and avoided the anti-obesogenic effect of in vivo TF administration.

**Conclusions:**

ILK downregulation in WAT can be considered a biomarker of obesity establishment. Via an INTB1-ILK axis, TF restores malfunctioning hypertrophied WAT by changing the expression of adipocyte-related genes, increasing ILK expression and activity, and reducing TG storage. TF prevents obesity, a property to be added to its anticoagulant and cardiovascular protective advantages.

**Supplementary Information:**

The online version contains supplementary material available at 10.1186/s13578-022-00746-1.

## Background

Obesity is a chronic disease related to white adipose tissue (WAT) that increases the risk of metabolic disorders such as diabetes mellitus type 2, non-alcoholic fatty liver disease, atherosclerosis, and arterial damage. The dysfunctional AT is unable to efficiently process the circulating metabolites and hyperlipidemia and/or hyperglycemia lead other organs to malfunction [1]. Under physiological conditions the adipocytes properly manage the uptake and disposal of triglycerides (TG) through lipogenic and lipolytic mechanisms. AT function is sustained by the continuous adipogenesis or the differentiation of pre-adipocytes to adipocytes [2]. During obesity, WAT depots enlarge by either the increase of the adipocyte number, known as WAT hyperplasia, or the shape rearrangement of the volume of pre-existing adipocytes by the increasing TG formation (lipogenesis) and storage in lipid droplets (LD), known as hypertrophy. The adipocytes’ differentiation state can be tracked by the expression and activity of biomarkers, such as the pro-inflammatory cytokines Monocyte Chemoattractant Protein-1 (MCP1) and Interleukin-6 (IL6); adipogenesis and differentiation genes, such as Peroxisome Proliferator-Activated Receptor-gamma (PPARγ) and the adipokine Leptin; beige/brown adipocyte marker Uncoupling Protein-1 (UCP1); proliferating cell nuclear antigen (PCNA); lipid metabolism enzymes, such as Fatty Acid Synthase (FASN), Hormone Sensitive Lipase (HSL) and Adipose Triglyceride Lipase (ATGL); and TG metabolite transporters, such as Fatty Acid Binding Protein 4 (FABP4), Fatty Acid Translocase (FAT) and Aquaglyceroporin 7 (AQP7). The expression of these biomarkers by the adipocytes, as well as the quantity and quality of extracellular matrix (ECM) surrounding them are changed in the malfunctioning WAT, [3–14]. To orchestrate ECM components with downstream intracellular architecture, all cells have adhesion receptors called integrins (INT), which are transmembrane heterodimers consisting of two subunits α and β [15]. INT subunits have been involved in obesity and insulin resistance [16, 17], particularly Integrin β1 (INTB1), which is the main WAT subunit and has been profusely studied as a final determinant of adipocyte differentiation [18–21]. INT1B activity is controlled by conformational structure switches and a bidirectional outside-in and inside-out signaling. INTB1 inside-out activation is initiated through the specific phosphorylation in T788/T789 of the cytoplasmic domain by components of the intracellular protein cluster, known as adhesome or interactome, and a requisite to conformationally change INTB1 from low-affinity to high-affinity binding with the ECM substrate and the facilitation of the outside-in signaling [22]. During the development of obesity, erratic ECM-INTB1 interaction causes WAT to malfunction through the remodelling of the adipocyte’s intracellular skeleton, which changes in LDs configuration and metabolites trafficking processes [23–27]. However, few publications have noted that ECM-based molecules may be used as a therapeutic strategy [5, 9, 17]. INTB1 lacks kinase activity, but several downstream intracellular kinases recruited in the interactome may be involved during the communication process, although the hierarchy and relevancy of each is not completely defined. Some of these kinases are protein kinase B (also known as AKT), glycogen synthase kinase 3β (GSK3β) or focal adhesion kinase (FAK) [28–31]. A major scaffold protein of the interactome recruited during INTB1 modulations is Integrin Linked Kinase (ILK) [32]. The role of ILK in obesity is relatively understudied. Some publications have timidly highlighted changes in ILK during adipogenesis in stem cells [33–35]. Few recent publications, including ours, have described ILK as a mediator during insulin-resistance and WAT remodeling in cells and mice models [36–38]. Most INT-target drugs are based on the amino acid consensus motif Arg-Gly-Asp (RGD), featured in ECM proteins and disintegrins from snakes venom [39]. Some competitively block fibrinogen binding to the INTs which are present in the platelets, and thus they are clinically used as an anticoagulant in patients with acute coronary syndromes undergoing percutaneous coronary intervention. The non-peptidic INT blocker Tirofiban (TF, Aggrastat) was the first molecule used for this purpose. Other INT-blockers are peptide-based, such as Eptifibatide (EPT), the clinical-discontinued Cilengitide (CIL) or Arg-Gly-Asp-Ser (RGDS) [39, 40]. The study of the functionality of INT-targeted drugs on other types of cells apart from platelets is promising [41]. For example, some preclinical studies, including ours, found a wider cardiovascular protective use for TF, rather than its intrinsic anticoagulant properties. [42–50]. To our knowledge, a pharmacological anti-obesogenic approach has not been performed previously with any clinically approved INTB1-targeted drug. Here, we performed experimental approaches in cultured differentiated adipocytes, originating from C3H10T1/2 stem cells [51], with or without a pharmacological INTB1 blockade or the transgenic depletion of ILK. TF was administered systemically in wildtype (WT) and ILK knockdown (cKDILK) mice during the establishment of obesity based on a high-fat diet (HFD) [36]. We determined body and WAT weight gains, ILK expression and activity, and adipogenesis and lipid metabolism markers, as discerned from the cultured cell model. We demonstrated the use of TF as anti-obesogenic and defined the relevance of ILK in the process.

## Methods

### Adipocytes culture and differentiation

C3H10T1/2 preadipocytes (CCL-226, ATCC, Manassas, USA) [43] were grown on 6-well plates with DMEM (Sigma-Aldrich, St. Louis, MO, USA) and 10% fetal bovine serum (FBS, Thermo Fisher Scientific Waltham, MA, USA). 2 days after cells reached confluency, they were induced to differentiate with DMEM supplemented with 10% FBS and Glutamax (Thermo Fisher Scientific), 1 µM dexamethasone, 1 µM rosiglitazone, 0.5 mM 3-isobutyl-1-methylxanthine (IBMX, Sigma-Aldrich) and 5 µg/ml insulin (Merck, Sigma-Aldrich) for 48 h. Medium was replaced every two days for a total of 6–10 days with DMEM supplemented with Glutamax, 10% FBS and 5 µg/ml insulin. The 9th days from the beginning of the differentiation, adipocytes were deprived of supplementation for 24 h. C2C12 myoblasts (CRL-1772, ATCC) and monocytes line THP-1 (TIB-202, ATCC) were grown in DMEM or RPMI (Sigma-Aldrich), respectively, with 10% FBS. Treatments were added under deprived conditions for the indicated times.

### Adipocytes transient transfection with siRNAs

siRNAs transfection was achieved in adhered adipocytes as described [52, 53]. Briefly, fully differentiated adipocytes were plated at 70–80% confluence in 6 well plates. 24 h later, adhered adipocytes were transfected with 20 nM specific siRNAs against ILK (siILK, Santa Cruz Biotechnologies Inc., Dallas, TX) or scramble siRNAs with Metafectene (Biontex, Munich, Germany) overnight and allowed to recover with DMEM + 10% FBS for another 24 h. Then, cells were deprived for 24 h and treated for the indicated times.

### LD staining and TG quantification

Fully differentiated adipocytes were deprived, treated, washed, and stained after the indicated times with lipid dyes. AdipoRed (Lonza, Basel, Switzerland) was used as indicated by the commercial manual. Fluorimetry was quantified using a plate reader with 485 nm excitation/572 nm emission (VictorX4 from Perkin-Elmer) and values were normalized to total protein content per well, determined by DC-Protein Assay (Bio-Rad, Hercules, CA, USA). A filtered dilution of Oil Red O (Sigma-Aldrich, 0.3 g in 100 ml of 60% isopropanol) was used on cells fixed with 4% formaldehyde and pictures were taken using an inverted microscope.

### Free glycerol determination in adipocyte cultures

Concentration of excreted glycerol in the culture medium supernatant after the treatments was measured using a commercial assay kit (BioVision, Milpitas, CA, USA) according to the manufacturer’s instructions and normalized to total protein content of the attached cells.

### F-actin quantification in adipocytes

C3H10T1/2 were seeded on coverslips and differentiated into adipocytes. After treatments, cells were fixed with 4% formaldehyde, permeabilized with 0.05% Triton-X-100, and blocked with 2.5% bovine serum albumin. After washes, covers were incubated with 0.1 µg/mL Alexa 568-phalloidin (Molecular Probes, Thermo Fisher Scientific Waltham, MA, USA) washed and nuclei were stained (ProLong Gold, Thermo Fisher Scientific. Fluorescence was analyzed under a confocal microscope TCS-SP5 (Leica Microsystems, Wetzlar, Germany). Phalloidin intensity was quantified and normalized to nuclei fluorescence using ImageJ software (NIH, USA).

### Animal models

Animal experiments have been approved by the Institutional Animal Care and Use Committees from Universidad de Alcalá and Comunidad de Madrid (PROEX 230/16), in agreement with the guidelines established by the European Community Council Directives (2010/63/EU). Adult conditional ILK-deficient mice (cKDILK) were generated and subjected to diets as previously described [37]. Briefly, C57Bl/6 mice homozygous for floxed ILK flanked by loxP were crossed with BALB/c strain mice carrying a CMV-driven hydroxytamoxifen -inducible (TX, Sigma-Aldrich, St. Louis, MO, USA) CreER(T) recombinase gene globally expressed in all the tissues. 8-week-old male and females were injected intraperitoneal with 1.5 mg of TX or vehicle, once per day for 5 consecutive days. 3 weeks after the injections, tail DNA was genotyped by PCR with primers corresponding to excised ILK gene (CCAGGTGGCAGAGGTAAGTA) or to non-excised ILK (CAAGGAATAAGGTGAGCTTCAGAA). The TX-treated mice displaying successful depletion of ILK were termed cKDILK, and the VH-treated without ILK depletion were termed wildtype (WT). Age-matched WT and cKDILK males and females were divided randomly and fed for 2 weeks either with a High Fat diet (HFD, 60 kJ% fat, 8.46% sucrose; D12492 Ssniff Spezialdiäten, Soest, Germany) or the corresponding low fat standard diet (STD, 13 kJ% fat, 67 kJ% carbohydrates, 10% sucrose; Envigo Teklad Global Diet 2014, East Millstone, NJ, USA). Diets were given ad libitum during the time of the experiment. Each mouse had free access to water and was kept on a 12:12 h light:dark cycle at constant temperature of 21–23 °C. The food intakes were calculated by subtracting the mass of food left from the initial food supplied. Along the period of diet challenges, TF (50 microg/KG, i.p.) or vehicle (saline) was administered daily [50]. After the experimentation conclude, animals were maintained under fasting conditions for 16 h, weighted and euthanized with pentobarbital overdose. Body weight gains were calculated subtracting weights at the beginning from weights at the end of each experimental timeline. WAT depots and vastus lateralis were dissected and freshly processed or after preservation in RNAlater (Thermo Fisher Scientific, Waltham, MA, USA). TF pharmacokinetics determinations were performed in *Rattus Norvegicus* (n = 6) after a single i.p. bolus of TF 50 microg/Kg. TF serological concentrations were determined in blood samples collected along 2 h after bolus and processed by liquid chromatography–mass spectrometry (LC–MS/MS).

### Reverse transcription–quantitative polymerase chain reaction (RT-qPCR)

All products and equipment used were from Thermo Fisher Scientific. After the corresponding experiment, total RNA was extracted from cells or tissues collected from fasting mice. Equal amounts of RNA were transcribed to cDNA with HighCapacity cDNA RT Kit and 10 ng of cDNAs were amplified using kits for qPCR. TaqMan gene expression assays were used to quantify MCP1 (Mm00441242_m1), IL6 (Mm00446190_m1), PPARγ, (Mm00440940_m1), Leptin (Mm00434759_m1), FASN (Mm00662319_m1), FABP4 (00445878_m1), HSL (Mm00495359_m1), ATGL (PNPLA2, Mm00503040_m1), AQP7 (Mm00431839-m1), FAT (Mm00432403_m1), UCP1 (Mm01244861_m1), PCNA (Mm05873628_g1) and βactin (Mm01205647_g1). Amplification values were normalized to endogenous β-actin and relative quantification was determined with 2^ − ΔΔCT method. To quantify the non-excised ILK sequence between exons within the floxed area number 6 and 7 in WT and cKDILK specially designed primers (GGGCTCTTGTGAGCTTCTGT and GAGTGGTCCCCTTCCAGAAT) [29]. were determined with SYBR Green Master Mix and normalized to β-actin (GACGGCCAGGTCATCACTAT and CTTCTGCATCCTGTCAGCAA).

### Protein extraction and immunoblot analysis

Cells or tissues were homogenized in lysis buffer (10 mM Tris–HCl, pH 7.6; 1% Triton X-100; 1 mM EDTA; 0.1% sodium deoxycholate) supplemented with protease and phosphatase inhibitors (Complete and PhosSTOP, Roche, Basel, Switzerland). Protein concentrations were determined by DC-Protein Assay (Bio-Rad, Hercules, CA, USA). Equal amounts were separated on SDS–polyacrylamide gels and transferred to 0.2 µm- PVDF membranes (Bio-Rad, Hercules, CA, USA). Membranes were blocked, incubated with primary antibodies and secondary antibodies (Merck-Millipore, Billerica, MA, USA or Dako, Glostrup, Denmark) afterwards. Primary antibodies used were against AKT, P-AKT (Ser473), GSK3β, P-GSK3β (Ser9), FAK, P-FAK (Tyr397), HSL, P-HSL (Ser660) (Cell Signaling Technology, Danvers, MA, USA), INTB1, P-INTB1 (Thr788/9) (Abcam, Cambridge, United Kingdom), Tubulin, Actin or GAPDH (Sigma-Aldrich, St. Louis, MO, USA). Immunoblots were detected by chemiluminescence (Pierce ECL Western Blotting Substrate, Thermo Fisher Scientific Waltham, MA, USA) and imaged with ImageQuant LAS 500 System (General Electric Healthcare, Little Chalfont, United Kingdom). Densitometries were measured using ImageJ software (NIH).

### Statistical analysis

GraphPad Prism 5 Software was used to perform Student's t test for 2 groups and 1- or 2-way analysis of variance form more groups, followed by Bonferroni's post hoc tests. Differences in mean values were considered statistically significant at a probability level of less than 5% (p < 0.05). Power of the study was 80–85%, with a confidence level of 95%.

## Results

### TF reduces TG content in the LD and the expression of adipogenesis markers in cultured adipocytes

**Fig. 1:**
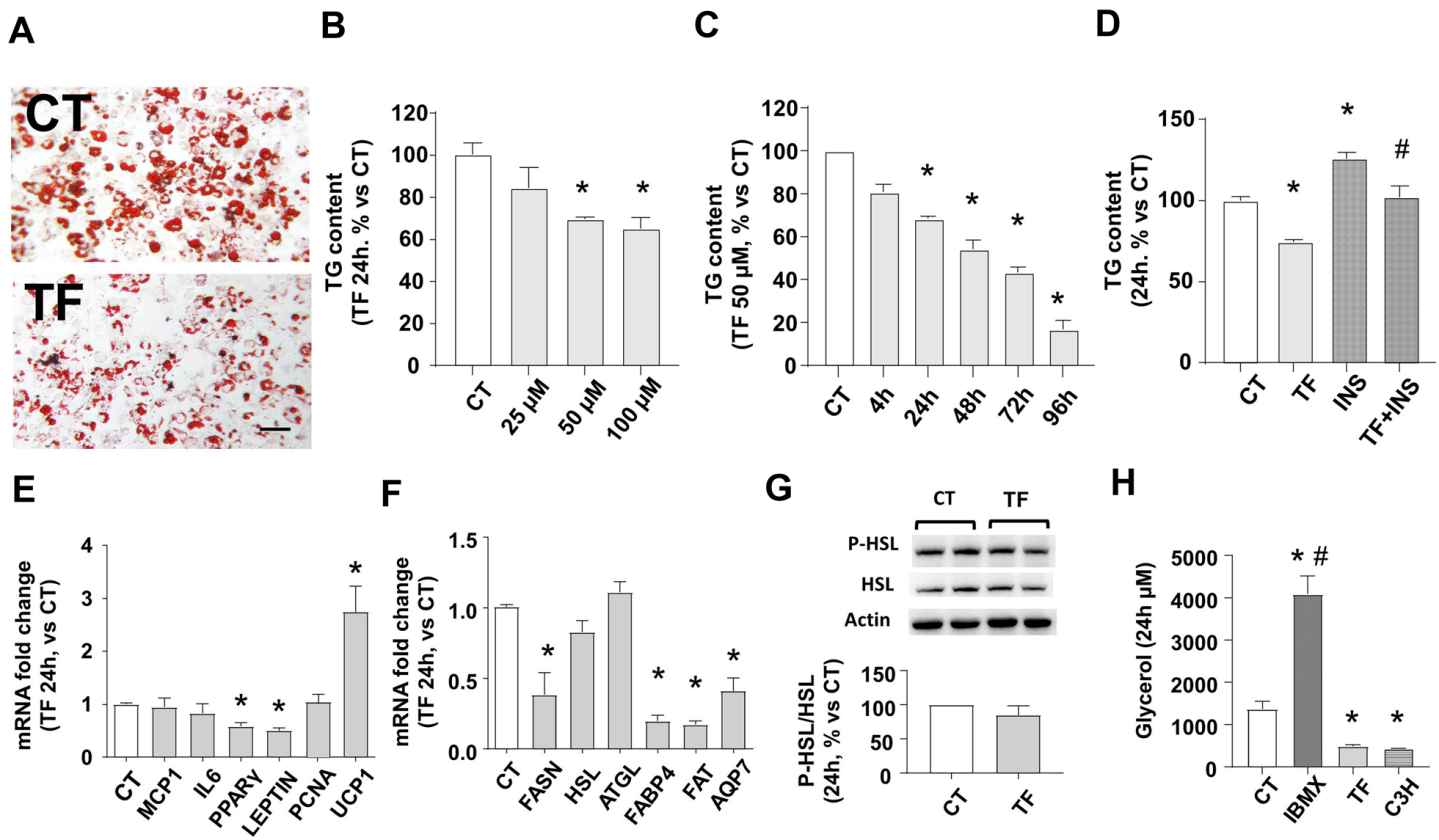
TF changes TG content and genetic pattern in adipocytes. Deprived differentiated adipocytes from c3H10T1/2 were treated with TF or vehicle (CT) at the indicated times and concentrations. **A** Representative image of LD stained with Oil-red O inside adipocytes after 24 h TF 50 µM. **B** Quantification of TG contained in LD, stained with AdipoRed after 24 h TF at different concentrations or **C** after different times with 50 µM TF or **D** after 24 h 100 nM insulin (INS) and TF 50 µM co-treatment. Insulin was added 15 min before TF or vehicle. **E** mRNA expression fold changes after 24 h TF 50 µM, analyzed by RT-qPCR and normalized to β-actin, of markers for inflammation (IL6 and MCP1), adipogenesis, (PPARγ, Leptin), proliferation (PCNA), browning (UCP1), and **F** lipid metabolism FABP4, FAT, AQP7, FASN, HSL, ATGL. **G** Representative immunoblots and densitometric analysis of phosphorylated HSL at Ser660 (P-HSL) vs total HSL protein contents; actin content is shown as endogenous control. **H** 24 h glycerol concentration in the supernatant secreted by cultured adipocytes treated with IBMX (0.5 mM), VH, TF or VH in undifferentiated C3H10t1/2 cells (C3H). Scale bars 50 μm. N = 8–12. Data are shown as % mean ± SEM vs CT. *p < 0.05 vs CT. #p < 0.05 vs TF

Adipoblast-like C3H10T1/2 cells, which are a model for adipogenesis commitment studies [51], were cultured to confluency and induced to differentiate with specific mediums replaced every 2 days, as detailed in the methods section, until the complete differentiation on the 9^th^ day. To avoid the biological effects from serum and other growth factors present in the culture mediums while performing the treatments, fully differentiated cells were serum-deprived 24 h before and during TF (Aggrastat, Tirofiban HCl 50 microg/ml, Correvio, UK) or vehicle (CT, physiological saline solution) treatments. TF concentrations used were in accordance with other in vitro experimental designs [47–49]. Figure 1 shows the quantification of TGs stored in LDs using lipid-specific dyes and the expression profile switch of some transdifferentiation markers after TF or CT treatment. Figure 1A shows representative microscopical pictures of adipocytes stained with the colorimetric lipidic dye Oil Red O. 50 microM TF reduces LD content per area after 24 h and modifies the apparent LDs distribution, from large LDs (as observed in CT) to smaller LD after TF treatment. Large LDs are characteristics of well differentiated adipocytes and WAT, meanwhile multilocular smaller LD are usually observed during transdifferentiation to non-adipogenic phenotype (pre-adipocyte, beige or brown adipocytes) in vitro [6]. Figure 1B shows LD content reduction of approximately 30% loss after 50 or 100 microM TF for 24 h, quantified with the fluorescent dye AdipoRed. Figure 1C shows that 50 microM TF-dependent LD content reduction appears as early as 4 h and significantly starts at 24 h. The LD content reduction was accumulative reaching the lowest value (approximately 70% loss) after 96 h. From this point onward, we assessed for the rest of the in vitro experiments the use of 50 microM TF for 24 h as the minimal TF functional conditions. Figure 1D shows the TG quantification in 24 h serum-deprived adipocytes co-treated with TF and 100 nM insulin for 24 h, as a comparative study of serum deprived-cells versus a physiological-like condition. Insulin naturally promotes adipogenesis by increasing TG storage and reducing its expense by lipolysis [2]. Insulin stimulation in CT increases the LD content after 24 h, as expected. However, TF prevented the insulin-induced LD content increase, reaching CT values. Increased pro-inflammatory adipocytokines, adipogenesis markers and intracellular TG content, are related with white adipocyte hypertrophy [2–5]. Figure 1E shows that TF treatment in cultured adipocytes does not modify the mRNA expression of pro-inflammatory adipocytokines MCP1 and IL6, while reducing adipogenesis markers (PPARγ and Leptin). TF so far inhibits hypertrophic signaling and does not affect WAT proliferation and hyperplasia, according to the unchanged expression of its marker PCNA [7]. TF increased UCP1, the thermogenic marker which is increased during white/hypertrophied to beige/brown adipocyte transdifferentiation [8–12]. Other genes that are altered during adipocyte hypertrophy are fatty acid synthase (FASN), the lipolytic enzymes HSL and ATGL, and TG metabolite transporters FABP4, FAT and AQP7 [3, 13, 14]. Figure 1F shows that TF downregulated FASN, FABP4, FAT and AQP7, but did not change HSL and ATGL. We further studied HSL activity and secreted glycerol to better study lipolysis during TF treatment. Figure 1G shows that 4 and 24 h of TF did not affect HSL phosphorylation at the crucial activation site Ser660. Secreted glycerol levels are shown in Fig. 1H. As a positive control, a single bolus of 0.5 mM IBMX, which activates the PKA-dependent canonical lipolytic pathway, increases the secretion of glycerol in our settings. By contrast, neither VH nor TF-treated cells were able to increase glycerol. This is in accordance with the unaltered lipases expression and activity shown in the previous panels. Moreover, the steady state levels of glycerol secreted by TF-treated cells was even smaller than VH-treated ones, reaching the low levels of glycerol from the undifferentiated C3H10t1/2. The lower-than-VH glycerol levels caused by TF emphasize its undifferentiating effect, which is probably due to the confluence of reduced TG content in the LD, reduced lipogenesis (FASN levels), unaltered lipolysis (lipases expression and activity), and reduced glycerol efflux (AQP7 levels).

### TF binds to INT1B, allowing rapid intracellular phosphorylation and the rearrangement of the actin cytoskeleton

**Fig. 2:**
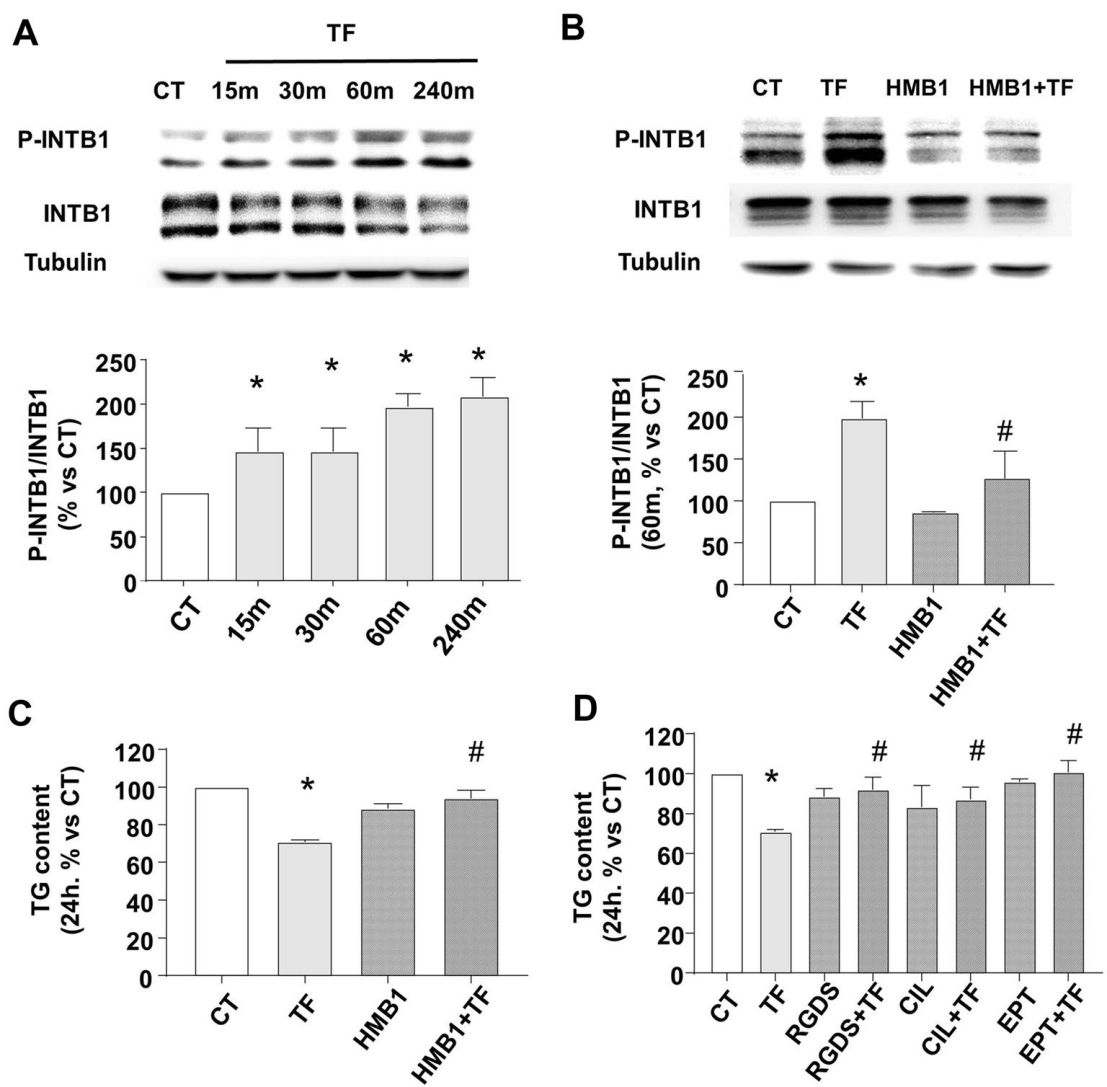
TF interacts with INTB1 to reduce TG content in adipocytes. Deprived differentiated adipocytes from c3H10T1/2 were treated with TF 50 µM or vehicle (CT) or co-treated with a specific INTB1 blocking antibody (HMB1), RGDS, eptifibatide (EPT) or cilengitide (CIL) for the indicated times. **A**, **B** Representative immunoblots and densitometric analysis of phosphorylated INTB1 at Thr788/9 (P-INTB1) vs total INTB1. Tubulin content is shown as endogenous control **C**, **D** quantification of TG contained in LD, stained with AdipoRed. N = 8–12. Data are shown as mean ± SEM, *p < 0.05 vs CT, #p < 0.05 vs TF

Figure 2A shows a rapid and significant INTB1 Thr788/9 phosphorylation [22] after TF treatment, starting at 15 min and maintained for at least 4 h. Although TF was able to rapidly modify the phosphorylation of INTB1, its expression, determined as mRNA and protein content, was not modified 24 h later (Additional file 1: Figure S1A and B). To study the specificity of TF binding to the extracellular domain of INTB1, cells were pre-incubated with a very specific function-blocking INTB1 antibody (HMB1, Anti-mouse CD29 clone HMBeta1-1) prior to the treatment with TF or vehicle. This antibody antagonizes INTB1 efficiently during the outside-in mediation of other ligands [54]. Figure 2B shows that INTB1 blockade with HMB1 was able to avoid 60 min TF-mediated INTB1 phosphorylation. Figure 2C shows that HMB1 blocked the functional TF-mediated reduction of TG content 24 h later. Moreover, Fig. 2D shows that outside-in blocking tetrapeptide RGDS and -fiban drugs EPT and CIL were as efficient as HMB1 in blocking LD reduction mediated by TF-INTB1.

**Fig. 3:**
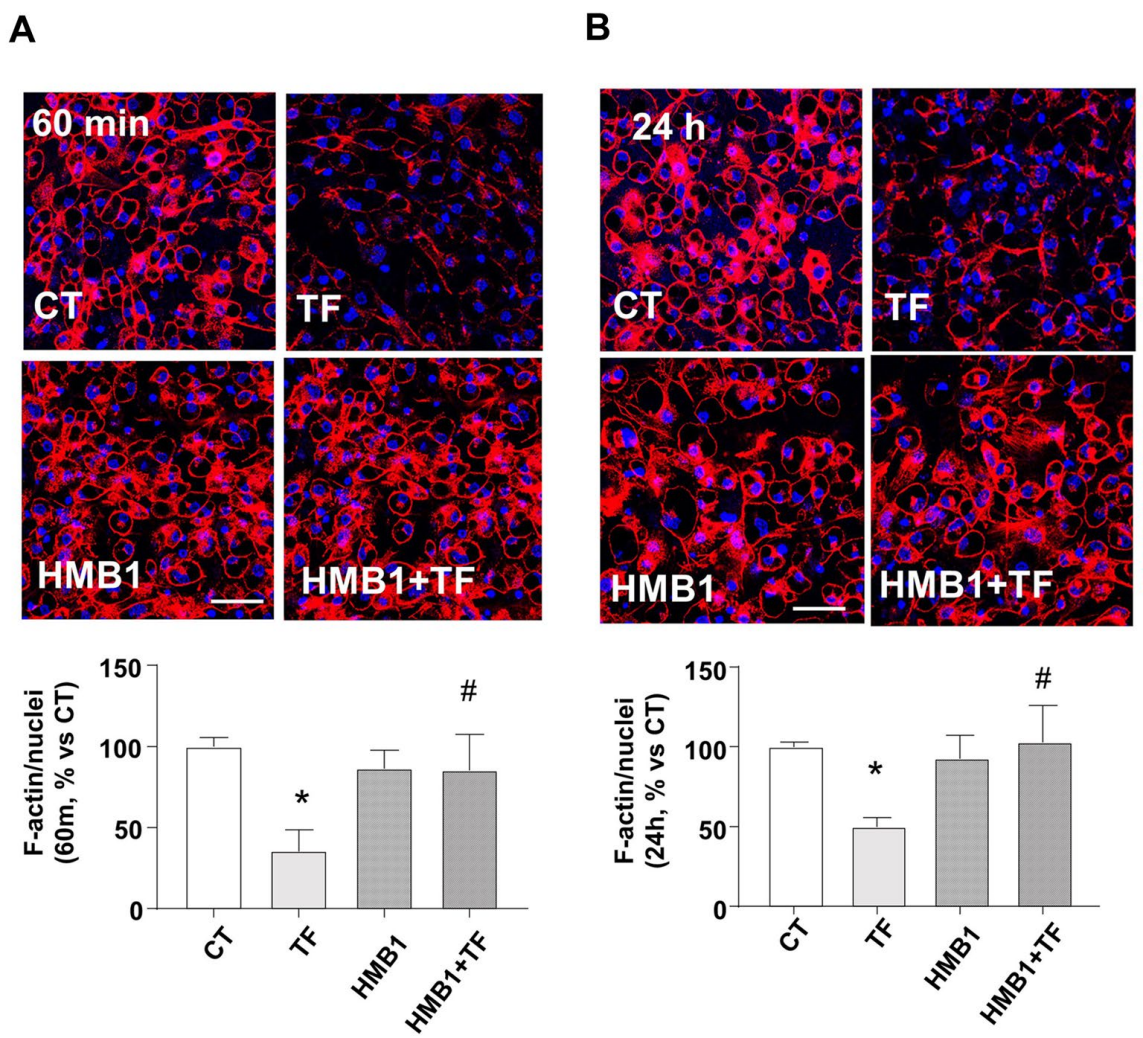
TF reduces the levels of F-actin in adipocytes. Deprived differentiated adipocytes from c3H10T1/2 were treated with TF 50 µM or vehicle (CT) or co-treated with specific INTB1 blocking antibody (HMB1) for the indicated times. Representative confocal microscopy images of F-actin dyed with phalloidin (red) and nuclei with DAPI (blue) and quantification of F-actin/nuclei per image after **A** 60 min and **B** 24 h of treatment. Scale bars 50 μm. N = 8–12. Data are shown as mean ± SEM, *p < 0.05 vs CT, #p < 0.05 vs TF

We followed the TF-INTB1 downstream regulation of actin cytoskeleton remodeling. Figure 3 shows the quantification of polymerized filamentous actin (F-actin) detected with phalloidin dye and normalized to nuclei present in the microphotography. TF disassembles (depolymerizes) F-actin in as quick as 60 min (Fig. 3A) and is maintained for 24 h (Fig. 3B). The pretreatment with INTB1- blocking antibody HMB1 avoid the TF-dependent actin depolymerization. Conclusively, these results note TF as a modulator of INTB1-actin status, which can be antagonized by an INTB1 outside-in blockade.

### TF-INTB1-ILK axis: TF binding to INTB1 increases ILK expression and activity and modifies the expression of other markers to reduce TG content

**Fig. 4:**
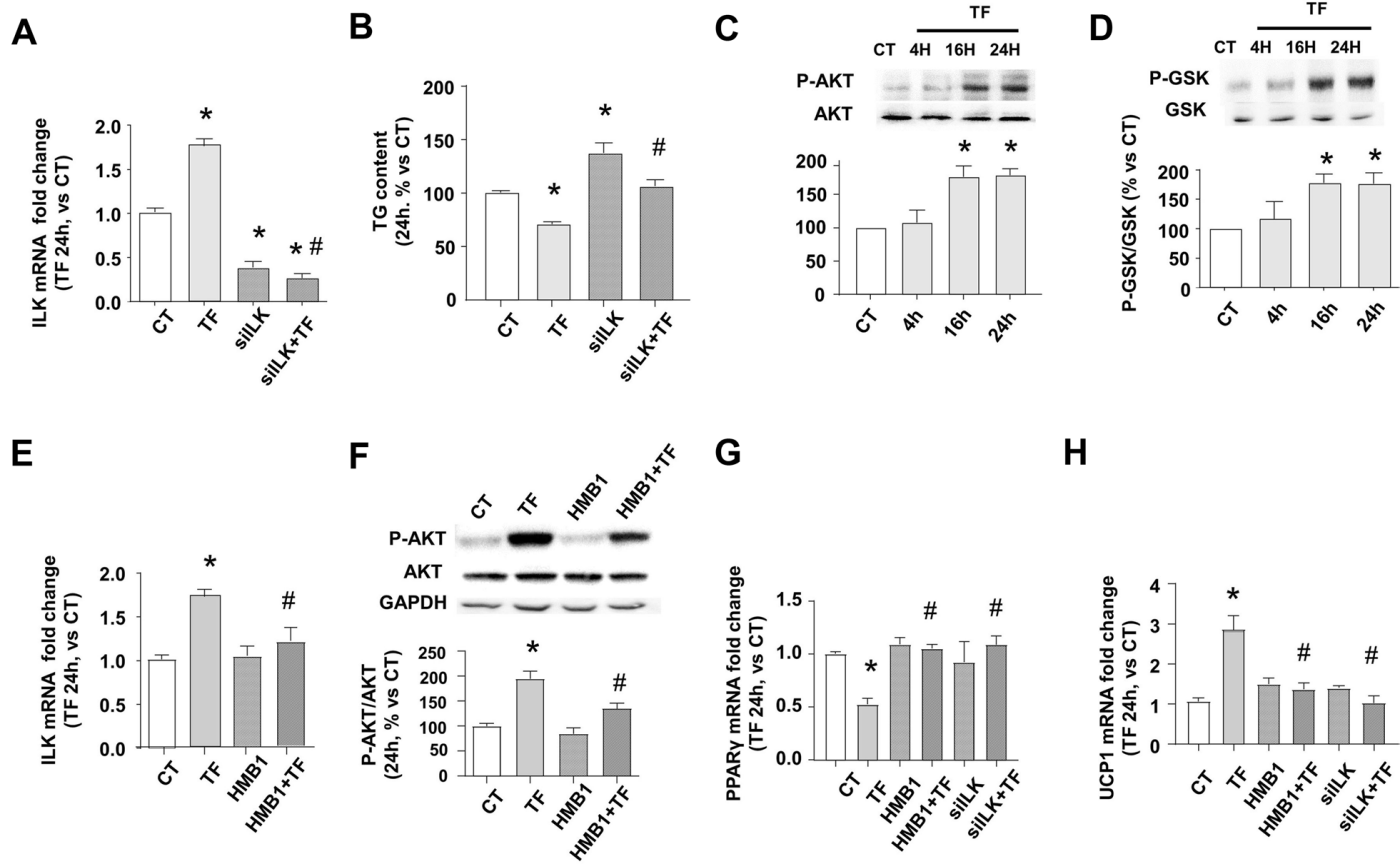
TF binding to INTB1 increases ILK expression and activity and modifies the expression of other markers to reduce TG content. Deprived differentiated adipocytes from C3H10T1/2 were transfected with specific siRNAs against ILK (siILK) or scrambled siRNAs as transfection control. 48 h after transfection, cells were deprived and treated with TF 50 µM or vehicle (CT) or co-treated with specific INTB1 blocking antibody (HMB1) at the indicated times. **A** Effect of 24 h TF and siILK transfection on ILK mRNA expressions fold change, analyzed by RT-qPCR and normalized to β-actin, and **B** TG contain stained with AdipoRed. **C** Representative immunoblots and densitometric analysis of AKT phosphorylated at ser 473 (P-AKT) and **D** GSK3β phosphorylated at ser 9 (P-GSK), normalized to the content of total AKT or GSK3β proteins respectively from control cells treated with TF 4, 16, 24 h or vehicle 24 h. **E** effect of 24 h TF and HMB1 on ILK mRNA expressions fold change, analyzed by RT-qPCR and normalized to β-actin and **F** effect of 24 h TF and HMB1 on P-AKT; representative immunoblots and densitometric analysis normalized to total AKT. **G** Effects of TF, siILK and HMB1 on adipogenesis marker PPARγ or **H** browning marker UCP1, analyzed by RT-qPCR and normalized to β-actin. N = 8–12. Data are shown as mean ± SEM, *p < 0.05 vs CT. #p < 0.05 vs TF

ILK is a main scaffold protein linking INTB1 and actin, and we studied its relevancy during TF-mediated transcriptional and functional. We blocked INTB1 and ILK pathways prior to TF treatment, by using the INTB1 blocking antibody HMB1 or ILK knockdown cells. To perform ILK knockdown in vitro, we transfected specific silencing RNAs (siRNA) against ILK (siILK). To assure that all the cells were under the same conditions, we used the same transfection conditions in all the experimentations shown in Fig. 4, with either control scrambled siRNA or siILK, as detailed in the methods section. Briefly, 48 h post-transfection, the cells were serum-deprived and treated with either HMB1 or vehicle 15 min before TF or vehicle treatments for 24 h. Figure 4A shows that TF increases the expression of ILK in cells transfected with scrambled siRNAs. The ILK depletion in siILK was only partial in both vehicle and TF treated cells, reaching around 70% depletion. The partial knockdown of ILK by siRNAS transfection has been previously reported as common in mature adipocytes [52, 53]. Figure 4B shows the consequences of ILK blockade on TG content. Cells transfected with scramble siRNAs and treated with TF have a similar TG downregulation as the non-transfected cells treated with TF, as shown in Fig. 1. Interestingly, the transfection with siILK significantly increased TG content compared to the control counterparts, which emphasizes the importance of the presence of ILK during the LD hypertrophy. On the other hand, TF was able to partially revert the TG increase caused by the partial depletion of ILK. We confirmed that ILK activity was increased in accordance with its expression after TF treatment, as measured by the phosphorylation state of well-known ILK downstream effectors AKT and GSK-3β [32]. Phosphorylation of AKT (Fig. 4C and Additional file 1: Figure S1C) and GSK-3β (Fig. 4D and Additional file 1: Figure S1D) were unaltered between 15 min to 4 h, the time period of the fast INT phosphorylation and actin depolymerization is shown in Figs. 2 and 3. However, TF was able to increase the phosphorylation of AKT and GSK-3β after a longer time of exposure, beginning as early as 16 h and maintained at 24 h, while the total content of both proteins was not affected. Figure 4E shows that INTB1 blockade with HMB1 partially avoid the TF-mediated increase of ILK expression. TF-dependent ILK activity shown as phosphorylation levels of AKT in Fig. 4F, was increased and partially blunted after INTB1 blockade, in accordance with the ILK expression levels. Furthermore, we studied the role of the INTB1-ILK axis driven by TF during the transcription of transdifferentiation genes, as already shown in Fig. 1. The blockade of INTB1 and ILK revert completely the TF-mediated expression changes on some representative markers, as PPARγ (Fig. 4G) and UCP1 (Fig. 4H). The effect on transcription was complete, unlike the partial effects observed during LD content (Fig. 4B) and ILK expression (Fig. 4E). Conclusively, TF uses INTB1-ILK axis to reduce TG content and to change the transcription of related genes. The axis blockade partially reverts the effect on TG content and ILK expression, but completely revert the transcription of other markers. The incomplete TF-mediated reversion of LD content in siILK-transfected cells can be interpreted as the participation of another ILK-independent mechanism, but it can be also considered the non-depleted ILK presence in siILK cells, where approximately 30% of ILK remains expressed and functionally modulated by TF to decrease partially the TG content. The partial transcriptional regulation of ILK when using HMB1 suggests the participation of a TF-INTB1-independent mechanism during ILK transcription, but not in the transcription of PPARγ and UCP1. Besides ILK, another kinase that is noted to be downstream INTB1 is FAK [31, 55]. TF does not modify FAK protein content nor its activity, which is measured as Tyr297 phosphorylation (Additional file 1: Figure S1E).

### In vivo approach: TF systemic administration prevents obesity appearance in a HFD-based mice model, increases ILK and modifies other markers expressions in visceral AT

**Fig. 5:**
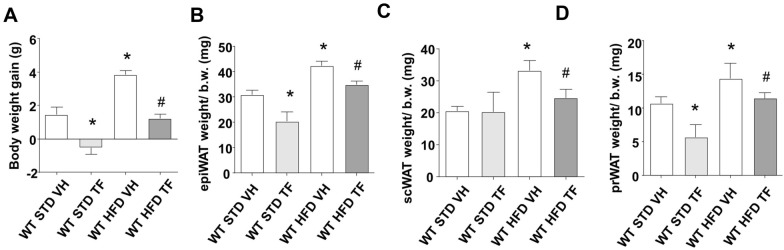
In vivo TF administration during HFD prevents obesity establishment and WAT weight gains. Control mice (WT) were challenged to a high-fat diet (HFD) or standard diet (STD) for 2 weeks and subjected to TF (50 microg/Kg/day, i.p.) or vehicle (VH). Afterward, animals were fasted overnight, weighed, sacrificed and WAT depots (WAT) were dissected. **A** total body weight (BW) gain over the 2 weeks challenge and **B** epididymal (epiWAT), **C** subcutaneous (scWAT) **D** perirenal (prWAT) WAT depots weights normalized to BW N = 6–12. *p < 0.05 vs WT STD VH, #p < 0.05 vs WT HFD VH

**Fig. 6:**
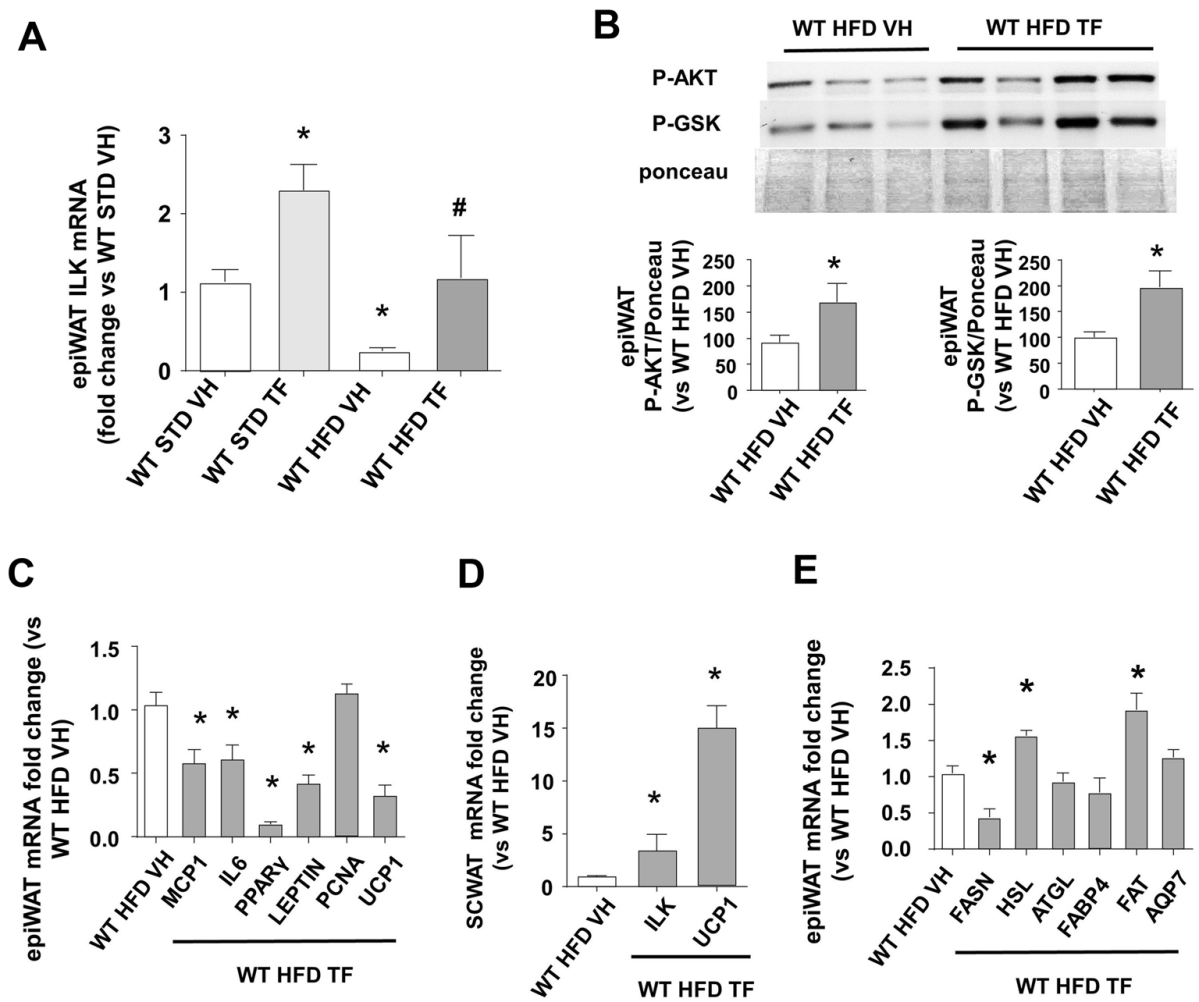
In vivo TF administration during HFD upregulates ILK in WAT and modifies the genetic pattern of several markers in WAT depots. Control mice (WT) were challenged to a high-fat diet (HFD) or standard diet (STD) for 2 weeks and subjected to TF (50 microg/Kg/day, i.p.) or vehicle (VH). Afterward, animals were fasted overnight, weighed, sacrificed and WAT depots (WAT) were dissected. **A** Fold changes of ILK mRNA expression analyzed by RT-qPCR and normalized to β-actin in epiWAT from STD and HFD WT. **B** Representative immunoblots and densitometric analysis of AKT phosphorylated at Ser473 (P-AKT) and GSK3β phosphorylated at Ser9 (P-GSK), normalized to total protein content (transference membrane dyed with ponceau) in epiWAT of HFD WT. **C** HFD WT epiWAT mRNA expression fold changes, analyzed by RT-qPCR and normalized to β-actin, of markers for inflammation (IL6 and MCP1), adipogenesis, (PPARγ, Leptin), proliferation (PCNA), browning (UCP-1). **D** HFD WT scWAT mRNA expression fold changes of ILK and UCP1. **E** HFD WT epiWAT mRNA expression fold changes of lipid metabolism markers FABP4, FAT, AQP7, FASN, HSL, ATGL. N = 6–12. *p < 0.05 vs WT STD VH, #p < 0.05 vs WT HFD VH

TF pharmacokinetics are similar in humans and rodents, with an early clearance of up to 2 h, and it is clinically approved as anticoagulant for a maximum of 18 h of continuous intravenous perfusion of 25 microg/kg. [56]. To observe functional effects in rodents, systemic administration of TF 50 microg/Kg body weight can be performed intraperitoneally (i.p.) [42–50]. In this regard, we first studied the pharmacokinetics of 6 rodents (*Rattus Norvegicus*) after a single i.p. bolus of TF 50 microg/Kg. To determine TF serological concentrations, blood samples were collected at different times and processed by LC–MS/MS. The maximum peak of TF was reached 15 min after i.p. and rapidly dropped to undetectable values after 2 h (mean values of TF in ng/ml of serum: time 0 control 0.39; 15 min 19.87, 30 min 15.73, 45 min 6.03, 60 min 6, 90 min 5.36, 120 min 0.51). To further elucidate the prolonged use of TF during the early establishment of obesity in vivo and the involvement of ILK, we designed a transgenic mice model [36, 37] with a global ILK downregulation during adulthood (cKDILK). These animals and their control counterparts (WT) without ILK depletion were divided to be challenged with either HFD or STD, the latter serving as control. HFD-challenged WT allowed us to follow TF consequences on the establishment of obesity, while HFD-challenged cKDILK are a transgenic tool to understand the relevance of ILK. We previously demonstrated that short-term HFD administration in mice for 2 weeks allows for the early establishment of obesity and insulin-resistance and for observation of ILK involvement [36]. Considering this and TF pharmacokinetics, we administered a single daily bolus i.p. of 50 microg/Kg TF per day in the mice throughout the 2 weeks of diet challenges. At the end of the experiments, mice were sacrificed, and WAT depots were dissected, weighed, and normalized to total body weight (BW). No difference in daily food or water intakes was observed between WT and cKDILK, nor between VH and TF-treated mice subjected to the same diet (Additional file 1: Table S1). The food grams intake was lower in VH HFD groups (WT or cKDILK) compared to VH STD counterparts, although the consumption of calories is increased due to HFD composition. Interestingly, food and water intakes of TF groups were not different to VH STD. Figure 5A shows BW gains between the beginning and the end of the diet/treatment challenge. HFD increased efficiently BW, despite the lower food intake, as we already published in our previous work [36]. Interestingly, TF treatment during this period reduced BW gain when compared to VH, in both STD and HFD challenged WT. Figure 5B–D, show, respectively, the weights of epididymal (epi), subcutaneous (sc) and perirenal (pr) WAT depots, normalized to BW, from the same animals. TF reduced epiWAT and prWAT ratios, but not scWAT in STD WT. All the WAT ratios were increased in VH-treated HFD WT, but were reduced in TF-treated HFD WT, in accordance with the BW gain changes shown in Fig. 5A. Figure 6A shows that TF increased ILK expression in epiWAT from both STD and HFD WT. INTB1 expression was unmodified in vivo and in vitro, while the phosphorylation in Thr788/9 did not persist after TF prolonged treatment in vivo, compared to the quick phosphorylation observed in vitro (Additional file 1: Figure S1F). The TF-dependent ILK upregulation in epiWAT from mice under physiological conditions and fed with STD mostly occurs in the main cell population present in WAT, which consists of adipocytes and pre-adipoctyes, although other cell types may be present to a lesser extent. However, under obesogenic HFD conditions, epiWAT is hypertrophied and the infiltrated macrophages population is increased, although the adipocytes-like population is the majority [1, 5, 6, 29]. We already published that ILK is downregulated in epiWAT from HFD [36] and TF was able to revert this to control (STD) values. Despite in vitro adipocyte ILK expression being upregulated by TF as in vivo (epiWATs from either STD or HFD), we wondered whether TF may change ILK expression in other cell types besides the adipocytes-like populations present mostly in WAT, such as macrophages, or even in other tissues, such as the skeletal muscle. Using the same conditions as with the cultured adipocytes, an in vitro approach of TF treatment was performed in macrophage cell line THP-1 and myocyte-like C2C12. TF was not able to change ILK expression in THP1 (Additional file 1: Figure S2A), C2C12 (Additional file 1: Figure S2B) and the skeletal muscle (vastus lateralis) from TF-treated HFD (Additional file 1: Figure S2C). Because therapeutic use of TF may be conceptually important in HFD hypertrophied WAT, we simplified the next panels by only exposing the results from HFD-challenged mice. Figure 6B shows that TF increases AKT and GSK3β phosphorylation in epiWAT from HFD. Figure 6C shows that TF reduces the mRNA expression of upregulated markers MCP1, IL6, PPARγ and leptin present in the hypertrophied epiWAT [1, 2, 23]. Proliferation marker PCNA was not modified. The transdifferentiation of beige adipocytes within WAT to brown-like phenotype is exhibited by higher levels of UCP-1, directly related to increased lipid metabolism and thermogenesis. [10]. Beige adipocytes exist mainly in scWAT rather than other visceral depots (e.g. epiWAT) in rodents [11, 12]. Figure 6C shows that TF did not increase UCP-1 in the epiWAT of HFD-mice, probably due to its limited browning capacity. We further studied in Fig. 6D the phenotypically more flexible scWAT inguinal depot, where ILK and UCP-1 were overexpressed after TF, which correlate with the trans-differentiation pattern observed in cultured adipocytes and suggesting a thermogenesis-mechanism in scWAT to lose energy and weight gain. On the other hand, epiWAT weight gain in HFD-mice is mostly caused by hypertrophy, thus increased lipogenesis and/or reduced lipolysis of white adipocytes [1–4]. Figure 6E shows that lipogenesis marker FASN was reduced in TF-treated mice, while lipase HSL was increased. ATGL, FABP4 and AQP7 remained unmodified, while FAT was increased. Altogether, these data indicate active lipolysis in epiWAT to reduce its weight gain.

### The lack of ILK in WAT avoids the anti-obesogenic effect of TF

**Fig. 7:**
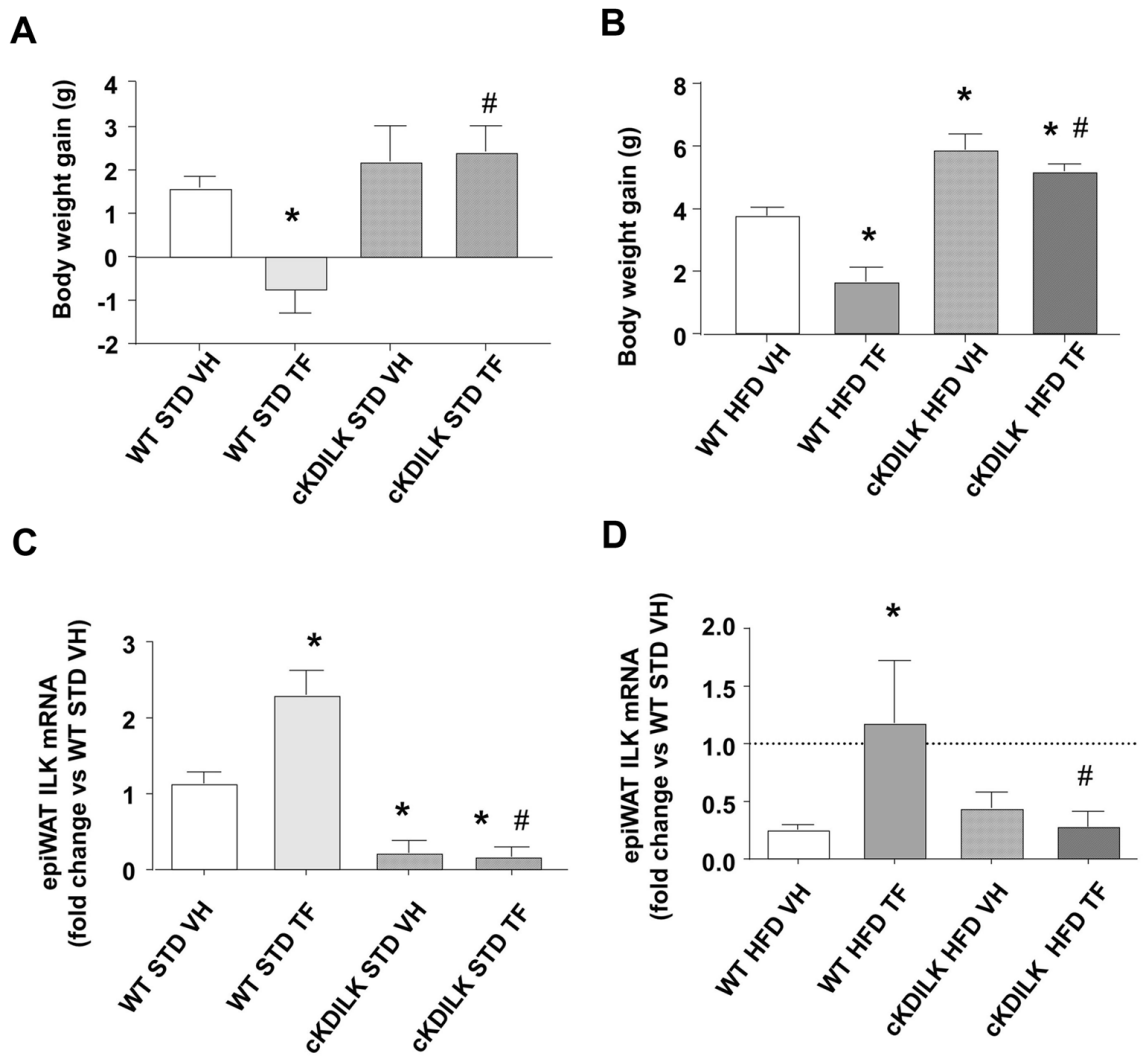
In vivo TF administration on ILK depleted cKDILK does not prevent obesity. Conditional Knockdown ILK (cKDILK) or control wildtype counterparts (WT) were challenged to a high-fat diet (HFD) or standard diet (STD) for 2 weeks and subjected to TF (50 microg/Kg/day, i.p.) or vehicle (VH). Afterward, animals were fasted overnight, weighed, sacrificed and epididymal white AT depots (epiWAT) were dissected, weighed, and processed accordingly. **A**, **B** total body weight (BW) gains over the 2 weeks challenge in STD and HFD, respectively. **C**, **D** fold changes of ILK mRNA expression analyzed by RT-qPCR and normalized to β-actin in STD and HFD, respectively. N = 6–12. *p < 0.05 vs WT VH, #p < 0.05 vs WT TF

**Fig. 8:**
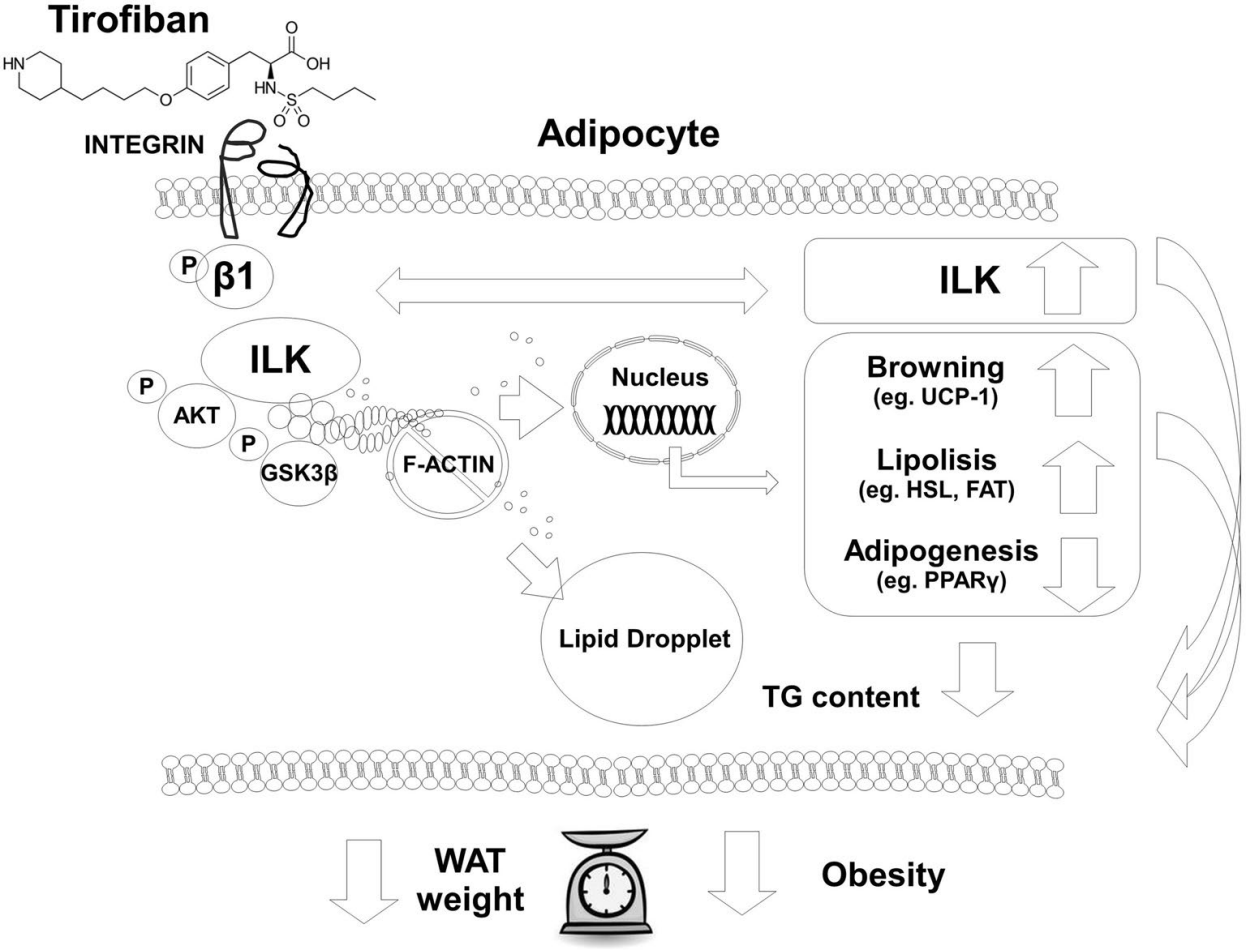
Putative mechanisms of TF-INTB1-ILK action. TF binds to adipocyte INTB1, depolymerizes F-actin and reduces the TG content on the lipid droplet. Adipogenesis-related genes are transcriptionally downregulated while lipolysis, thermogenesis and ILK genes are upregulated. ILK presence is part of the gear for TF function. Therefore, WAT adipocytes transdifferentiate to catabolic/beige phenotype to reduce WAT hypertrophy and obesity

We further studied the relationship between BW gains and ILK presence in epiWAT by using cKD-ILK challenged to diets and i.p. treatments as their WT counterparts. Figure 7A, B show BW gains of STD andr HFD-challenged animals, respectively. Under both diets, TF was not able to reduce BW gains in cKDILK as happens in WT. Moreover, Fig. 7B also shows that HFD cKDILK BW gain is even higher than that of HFD WT, something that has already been observed in our previous work [36]; however TF was not able to minimally decrease this BW gain. Figure 7C, D show the efficient ILK depletion in the epiWAT of cKDILK when compared with that of WT in both STD and HFD. The ILK expressions are in accordance with BW gains. In both cases, TF was not able to revert the ILK downregulation in cKDILK, while in TF treated WT, the increase of ILK expression was clearly shown (Fig. 5). Figure 8 summarizes a putative INTB1-ILK mediated mechanism of TF in WAT adipocytes. ILK presence is relevant during obesity establishment. TF pharmacologically transdifferentiates the adipocytes within hypertrophied WAT and therefore avoid obesity. TF uses the axis formed by INTB1-ILK-actin in the adipocytes to change lipidic metabolism, intracellular TG content and adipocytic and ILK genes transcription.

## Discussion

Obesity is one of the major global health concerns, even though only a few medications have been approved for the reduction of energy intake, its absorption or the promotion of its expenditure. Due to the long-term side effects, particularly cardiovascular issues, new anti-obesity drug development must be focused on both weight loss efficacy and cardiovascular safety [50]. In this study, we demonstrated the unexpected use of clinically approved TF as a potential anti-obesogenic. TF is particularly relevant as cardiovascular protector because it is an anticoagulant with vasodilator and endothelial protective properties [42–50]. Moreover, other RGD-based “-fiban” drugs were developed as vascular protective “superaspirines” [39, 40]. Our approach has never been considered for other accepted anti-obesogenic drugs: TF reduces adipogenesis in both cultured adipocytes and hypertrophied WAT from diet-induced obese mice. To target INTB1 signaling appears to be a logical method to alter adipogenesis and prevent obesity, since hypertrophic adipocytes have upregulated levels of INTB1 and there is an intimate relation between INTB1 and adipogenesis [5, 18–21]. However, almost no attention had been given to INT1B modulation in AT malfunction until the development of this study, although the potentiality of these agents as therapeutics in other applications is patent [9]. The importance of INTs during the development of adipose tissue has been emphasized with the use of transgenic knockout mice models. However it is not a good approach for the study of obesity. Indeed, the complete knockout of INTB1 is lethal [18] and the adipose-selective conditional deletion of INTB1 produces an atypical lipodystrophy [19]. On the other hand, the knockout of INT subunit alpha4, an INTB1 partner in adipocytes, can ameliorate HFD-based obesity [16]. In accordance with these premises, here we demonstrated that the pharmacological interaction of TF specifically with INTB1 ameliorated obesity, and the use of known outside-in blockers such as HMB1, RGDS, CIL and EPT [39, 40, 54] blocked the effect of TF while they were not able to reduce TG content by themselves. TF interacted with INTB1 and produced rapid phosphorylation of cytoplasmic Thr788/9, which is part of inside-out rather than outside-in signaling [22], but due to the fine-tuned balancing act between inside-out structural modulation and outside-in functional modulation, further research may be devoted to understanding the rapid phosphorylation of INTB1 as well as which adaptor proteins are involved during this early inside-out signaling. We found some of the mechanisms followed by the TF-INTB1 interaction, however, we did not completely list the sequence of events, because adipocyte transdifferentiation occurs due to an intimate and not well-understood relationship between cytoskeletal rearrangement, TG storage/expenditure, LD configuration (size, locularity, morphology), metabolite transport dynamics and transcriptional regulation [15, 23–27]. In vitro, TF reduced LD size, increased multilocular LD configuration and the expression of pre-adipoctye and/or beige adipocyte markers, in accordance with anti-hypertrophic transdifferentiation [19]. In WAT from animals treated with TF, transdifferentiating mechanisms of lipolysis and/or browning were also patent. It is not easy to track and interpret the exact process of communication between TF-INTB1 and the downstream cytoskeletal rearrangement, because the modulation of INTB1 during WAT differentiation or dysfunction is highly complex, not linear and involves a vast array of enzymes, adaptors, and cytoskeletal components. [5, 8, 9, 18–21]. Several inconsistencies are reported concerning the remodeling and dynamics of each cytoskeleton component (e.g., actin, microtubules) during adipocyte maturation, due to variable experimental conditions (e.g. cultured cell model, AT depots used from animal models, intracellular location of the network, etc.) [24, 27]. For example, adipocyte hypertrophy correlates with increased F-actin in some of these reports [20, 25] and its disruption reduces adipose differentiation [57], while other reports suggest opposite observations, that is, adipogenesis is enhanced when cytoskeleton components are disrupted [26, 58–60].

The use of INTB1-blocking agents demonstrated that TF-mediated rapid and persistent actin depolymerization is part of the specific INTB1 outside-in signaling started by TF in correlation with the reduction of LD content. TF reduced F-actin levels in vitro, but that does not necessarily mean that the cytoskeleton is completely disrupted, unestablished, or that its dynamic is blunted. Actin depolymerization mediated by TF which extends from the cytoplasm to the nucleus, may be the mechanical base for the transcriptional modulation during adipocyte differentiation, as demonstrated in several cell types [61, 62]. Our work is distant to completely clarify TF-driven transmission between actin and the nuclear transcription. Nevertheless, actin depolymerization by TF is expected to be followed by the adipocytes within WAT, as demonstrated by comparative studies of cultured adipocytes vs whole tissue WAT [63]. We used two experimental approaches to address our hypothesis, a single dose of TF in cultured adipocytes and HFD-based obesogenic model in vivo with systemic TF administration for several days. The evident differences may result in differences of expressions and mechanisms followed inside the adipocytes. TF did not increase pro-inflammatory cytokines expression in vitro, an expected safety concern already addressed since TF is an approved drug. Interestingly, TF reduced these cytokines in WAT from in obesogenic (HFD) mice, which gives an advantageous effect of TF by reducing WAT inflammation that causes malfunction [1, 5]. Adipocyte hypertrophy is upregulated during obesogenesis and can be tracked by the expression of PPARγ and leptin, which were both blunted by TF. TG content (LD size) reconfiguration in vitro may be based on reduced lipogenesis rather than increased lipolysis: a) TF reduces LD content despite the stimulation of anti-lipolytic insulin [2], b) lipogenesis marker FASN was reduced and b) neither the expression of lipases, nor HSL activity [4] were increased in cultured adipocytes treated with TF, while the pivotal glycerol efflux channel AQP7 [13, 14] and therefore the steady state glycerol secretion outside the cells were downregulated. Moreover, the TF-mediated lower-than-VH basal steady-state glycerol secretion reached undifferentiated C3H10T1/2 levels, which also may indicate the grade of undifferentiation reached by TF-treated adipocytes. On the other hand, long term in vivo use of TF increased the expression of lipolysis markers HSL and FAT in epiWAT. Therefore, it is plausible that TF uses lipolysis in vivo, as part of the mechanisms to reduce hypertrophic visceral WAT weights in HFD [1, 2, 4]. TF increased thermogenic UCP1 [8–12] while decreased AQP7 in vitro, pointing a white-to-brown transdifferentiation. AQP7 downregulation is considered another beige adipocyte differentiation marker, because the heat production from oxidative metabolism related to the beige adipocytes contrasts with the anabolic/catabolic lipid metabolism requiring glycerol gateways occurring in white adipocytes [13, 14]. In TF-treated mice, UCP-1 expression was increased in scWAT, which is profuse in beige adipocytes [10–12], although UCP1 and AQP7 were not modified in epiWAT. Increased thermogenesis in scWAT was therefore the responsible pathway followed to produce the weight loss. Conclusively, food intakes were the same between HFD groups, but TF increases the expenditure of energy overload by rather increasing WAT depot-dependent lipolysis and/or thermogenesis. We found the relevant role of ILK during hypertrophy in adipocytes because ILK loss produces an aggravation of the hypertrophy inside the adipocytes and in the WAT weights. By using TF, we demonstrated that increase of ILK yield in WAT is protective against obesity, and the ILK upregulation was specific for adipocytes-like cells within the WAT and it was not observed in other TF-treated cell types and/or tissues. The blockade of INTB1 with HMB1 and the use of cells without ILK (siILK) demonstrate the importance of TF-INTB1-ILK transmission of the transcriptional and functional effects. INTB1 blockade was able to completely revert the TF-mediated TG content change, as well as the actin depolymerization. HMB1 completely revert TF-mediated PPARγ and UCP1 transcription while ILK upregulation was only partially reverted, probably due to the participation of different mechanisms (e.g., Transcriptional factors). ILK blockade (siILK) also reverted completely the TF-mediated transcription of PPARγ and UCP1, while the reversion of LD downregulation was partial, which can be interpreted as the participation of an ILK-independent mechanism, or it can be also considered the presence in siILK cells of approximately 30% non-depleted ILK, which remains functional and therefore can be modulated by TF to decrease only partially the TG content. We studied the ILK effects in vivo, by using a transgenic model of global ILK depletion in the adulthood (cKDILK). Our previous works showed that cKDILK under HFD, where ILK is successfully under-expressed in WAT, are predisposed to gain weight and insulin resistance [36, 37], and therefore can be considered a better tool than other knockouts for upstream or downstream elements. Together with the present work, our results support the hypothesis that ILK expression is decreased during the development of obesity and WAT transgenic downregulation of ILK (in cKDILK) exacerbates the obesity-related symptoms. The opposite hypothesis has been stated in Bugler-Lamb´s work [38], where ILK expression was increased by a long-term HFD and the use of adipocyte-specific ILK-deficient mice displayed reduced fat mass and improvement of glucose tolerance, as the in vitro knockdown of ILK in 3T3-L1 cells decreased lipid accumulation in their work. The differences of both methodological and conceptual approaches between Bugler-Lamb´s models and ours are several and may explain the apparently contradictory results between studies. Nevertheless, we did carry out a pharmacological strategy to increase ILK expression in vitro and in vivo as a protective approach against obesity that was not used in Bugler-Lamb´s study. The ILK upregulation is accompanied by the increased activity of ILK, measurable by the phosphorylation of downstream effectors such as AKT at Ser473 as well as GSK3β at Ser9. AKT/GSK3β phosphorylation, and by extension ILK activity, were not as rapid as INTB1 phosphorylation in vitro. Therefore, these kinases were probably not responsible for the rapid inside-out TF-mediated INTB1 phosphorylation. Other kinases have been noted as adaptor enzymes during adipocytic differentiation, such as mitogen-activated protein kinase/extracellular signal-regulated kinase, PI3K, Rho-associated protein kinase, AMP-activated protein kinase, and the Src kinases family [28–30]. Particularly, FAK is usually referred to as being placed upstream in the hierarchy of recruitment and activation of those kinases, including AKT during INTB1 modulation [31]. Our results showed that FAK activity, analyzed by phosphorylation at Tyr397, was not altered throughout the TF treatment time-lapse. Thus, it is probable that FAK or related downstream kinases are not relevant during the TF-mediated effects. Although ILK activity may not be responsible for upstream INTB1 phosphorylation, our results indicate that an axis made up of ILK-AKT/GSK3β may be responsible for the downstream TF-mediated reduction of adipogenesis and hypertrophy. Some publications agree with this hypothesis: AKT and GSK3β are related to adipocyte differentiation [28–30] and, as we already demonstrated, cKDILK animals have reduced AKT phosphorylation in epiWAT, concomitantly with the downregulated ILK expression and the increased fat mass and insulin resistance [36, 37] as well as TF depends on AKT to favor endothelial cell growth [42, 43]. AKT/GSK3β showed phosphorylation after a longer period of TF treatment (16/24 h in vitro, 2 weeks of daily administration in vivo), in accordance with the observed increased expression of ILK, however, it is also probable that the repeated and long-time exposure of TF in vivo produces an indirect effect on AKT/GSK3β. Nevertheless, to address the role of each above-mentioned kinases (besides ILK) during TF-mediated adipocyte transdifferentiation, further experimental models may be necessary (e.g. Specific silencing of each kinase).

## Conclusions

We consider ILK expression levels in WAT to be a biomarker of metabolic malfunction during the development of obesity. Pharmacological treatments able to increase ILK expression are a new strategy to restore the malfunctioning WAT. TF is a well-established anticoagulant with known cardiovascular protective benefits. TF increases ILK expression and activity and reduces adipogenesis and facilitates adipocyte transdifferentiation. The consequence is that TF administration in vivo reduces WAT hypertrophy and weight gain. These preclinical results provide the proof of concept for more translational studies to design a cardiovascular safe treatment based on TF-mediated interaction of INTB1-ILK.

## Supplementary Information

**Additional file 1: Table S1.** Food and water intakes during experimental conditions in vivo. Conditional Knockdown ILK (cKDILK) or control wildtype counterparts (WT) were challenged to high fat diet (HFD) or standard diet (STD) for 2 weeks and subjected to TF (50 microg/Kg/day, i.p.) or vehicle (VH). Food and water intakes per animal were measured every day along the experiment. Values are represented as mean + / − SEM. N = 6–12. *p < 0.05 vs STD. **Figure S1. **TF does not modify INTB1 and FAK expressions after 24 h, phosphorylation of AKT, GSK and FAK from 15 to 240 min, FAK at 24 h or INTB1 after 2 weeks administration in vivo. **A**–**E** Deprived differentiated adipocytes from c3H10T1/2 were treated with TF 50 µM or vehicle (CT) for the indicated times. A) 24 h fold changes of INTB1 mRNA expression were analyzed by RT-qPCR and normalized to β-actin B), Representative immunoblots and densitometric analysis of total INTB1 levels after 24 h, normalized to tubulin levels. **C**, **D** Representative immunoblots and densitometric analysis of AKT phosphorylated at Ser473 (P-AKT) and GSK3β phosphorylated at Wer9 (P-GSK) respectively, between 15 and 240 min and normalized to total AKT or GSK3β protein contents. **E** Representative immunoblots and densitometric analysis of FAK phosphorylated at Tyr297 (P-FAK) between 15 min and 24 h and normalized to total FAK protein content. **F** Control mice (WT) were challenged to high fat diet (HFD) for 2 weeks and subjected to TF (50 microg/Kg/day, i.p.) or vehicle (VH). Afterward, animals were fasted overnight, weighed, sacrificed and epididymal white AT depots (epiWAT) were dissected and processed. Representative immunoblots and densitometric analysis of phosphorylated INTB1 at Thr788/9 (P-INTB1) vs total INTB1. Data are shown as mean ± SEM. N = 6–12. **Figure S2.** TF does not modify ILK expression in monocytes in vitro or in skeletal muscle in vitro and in vivo. A) Deprived cultured monocytes THP1 and B) myoblasts C2C12 were treated with TF 50 µM or vehicle (CT) 24 h and ILK mRNA expression fold changes were analyzed by RT-qPCR and normalized to β-actin. **C** Control mice (WT) were challenged to high fat diet (HFD) for 2 weeks and subjected to TF (50 microg/Kg/day, i.p.) or vehicle (VH). Afterward, animals were fasted overnight, weighed, sacrificed and vastus lateralis were dissected. Fold changes of ILK mRNA expression analyzed by RT-qPCR and normalized to β-actin. Data are shown as mean ± SEM. N = 6–12.

## Data Availability

All data generated or analyzed during this study are included in this published article and its additional files.

## Abbreviations

AKT: Protein kinase B
AQP7: Aquaglyceroporin 7
ATGL: Adipose Triglyceride Lipase
BW: Body weight
CIL: Cilengitide
cKDILK: ILK knock-down mice
CT: Control, vehicle-treated cells
ECM: Extracellular matrix
epiWAT: Epididymal WAT depot
EPT: Eptifibatide
F-actin: Polymerized filamentous actin
FABP4: Fatty Acid Binding Protein 4
FAK: Focal adhesion kinase
FASN: Fatty Acid Synthase
FAT: Fatty Acid Translocase
FBS: Fetal bovine serum
GSK3β: Glycogen synthase kinase 3β
HFD: High fat diet
HMB1: Anti-mouse CD29 clone HMBeta1-1, INTB1 blocking antibody
HSL: Hormone Sensitive Lipase
IBMX: 3-Isobutyl-1-methylxanthine
IL6: Interleukin-6
ILK: Integrin-linked kinase
INS: Insulin
INT: Integrin
INTB1: Integrin-β1
LC–MS/MS: Liquid chromatography–mass spectrometry
LD: Lipid droplets
MCP1: Monocyte chemoattractant protein-1
PCNA: Proliferating cell nuclear antigen
prWAT: Peri-renal WAT depot
RGD: Arg-Gly-Asp peptide
RGDS: Arg-Gly-Asp-Ser peptide
scWAT: Subcutaneous WAT depot
siILK: Silencing RNA against ILK
siRNA: Silencing RNA
STD: Standard diet
TF: Tirofiban
TG: Triglycerides
TX: Tamoxifen
UCP1: Uncoupling Protein-1
WAT: White adipose tissue
WT: Wildtype mice
