# Supplementary material for: The integrin beta1 modulator Tirofiban prevents adipogenesis and obesity by the overexpression of integrin-linked kinase: a pre-clinical approach in vitro and in vivo
Source: Cell Biosci. 2022 Jan 28;12:10. doi: 10.1186/s13578-022-00746-1 (PMC8796419; doi:10.1186/s13578-022-00746-1)
Supplement: Supplementary file 1 — Additional file 1: Table S1. Food and water intakes during experimental conditions in vivo. Conditional Knockdown ILK (cKDILK) or control wildtype counterparts (WT) were challenged to high fat diet (HFD) or standard diet (STD) for 2 weeks and subjected to TF (50 microg/Kg/day, i.p.) or vehicle (VH). Food and water intakes per animal were measured every day along the experiment. Values are represented as mean + / − SEM. N = 6–12. *p < 0.05 vs STD. Figure S1. TF does not modify INTB1 and FAK expressions after 24 h, phosphorylation of AKT, GSK and FAK from 15 to 240 min, FAK at 24 h or INTB1 after 2 weeks administration in vivo. A–E Deprived differentiated adipocytes from c3H10T1/2 were treated with TF 50 µM or vehicle (CT) for the indicated times. A) 24 h fold changes of INTB1 mRNA expression were analyzed by RT-qPCR and normalized to β-actin B), Representative immunoblots and densitometric analysis of total INTB1 levels after 24 h, normalized to tubulin levels. C, D Representative immunoblots and densitometric analysis of AKT phosphorylated at Ser473 (P-AKT) and GSK3β phosphorylated at Wer9 (P-GSK) respectively, between 15 and 240 min and normalized to total AKT or GSK3β protein contents. E Representative immunoblots and densitometric analysis of FAK phosphorylated at Tyr297 (P-FAK) between 15 min and 24 h and normalized to total FAK protein content. F Control mice (WT) were challenged to high fat diet (HFD) for 2 weeks and subjected to TF (50 microg/Kg/day, i.p.) or vehicle (VH). Afterward, animals were fasted overnight, weighed, sacrificed and epididymal white AT depots (epiWAT) were dissected and processed. Representative immunoblots and densitometric analysis of phosphorylated INTB1 at Thr788/9 (P-INTB1) vs total INTB1. Data are shown as mean ± SEM. N = 6–12. Figure S2. TF does not modify ILK expression in monocytes in vitro or in skeletal muscle in vitro and in vivo. A) Deprived cultured monocytes THP1 and B) myoblasts C2C12 were treated with TF 50 µM or vehicle [file 13578_2022_746_MOESM1_ESM.docx]

**ADDITIONAL FILE 1**

**The integrin beta1 modulator Tirofiban prevents adipogenesis and obesity by the overexpression of integrin-linked kinase: a pre-clinical approach in vitro and in vivo.**

De Frutos S ^1, 2, 3, 4^*, Griera M ^1, 2, 3^, Hatem-Vaquero M ^1, 2^, Campillo S ^1, 2^, Gutiérrez-Calabres E ^1, 2^, García-Ayuso D ^1, 2^, Pardo M ^5^, Calleros L ^1, 2^, Rodríguez-Puyol M ^1, 2^, Rodríguez-Puyol D ^2, 4, 6^.

|  | **STD FOOD INTAKE**  **(g/24 h)** | **STD WATER INTAKE**  **(ml /24h)** | **HFD FOOD INTAKE**  **(g/24 h)** | **HFD WATER INTAKE**  **(ml/24)** |
| --- | --- | --- | --- | --- |
| **WT + VH** | 3.5 +/- 0.3 | 3.7 +/- 0.5 | 2.4 +/- 0.2 (*) | 4.0 +/- 0.8 |
| **cKDILK + VH** | 3.6 +/- 0.3 | 3.3 +/- 0.6 | 2.6 +/- 0.4 (*) | 3.5 +/- 0.4 |
| **WT + TF** | 3.1 +/- 0.3 | 3.6 +/- 0.3 | 3.3 +/- 0.4 | 3.6 +/- 0.2 |
| **cKDILK + TF** | 3.8 +/- 0.3 | 3.8 +/- 0.6 | 3.1 +/- 0.1 | 3.6 +/- 0.4 |

**TABLE S1: Food and water intakes during experimental conditions in vivo.** Conditional Knockdown ILK (cKDILK) or control wildtype counterparts (WT) were challenged to high fat diet (HFD) or standard diet (STD) for 2 weeks and subjected to TF (50 microg/Kg/day, i.p.) or vehicle (VH). Food and water intakes per animal were measured every day along the experiment. Values are represented as mean +/- SEM. N=6-12. *p<0.05 vs STD.


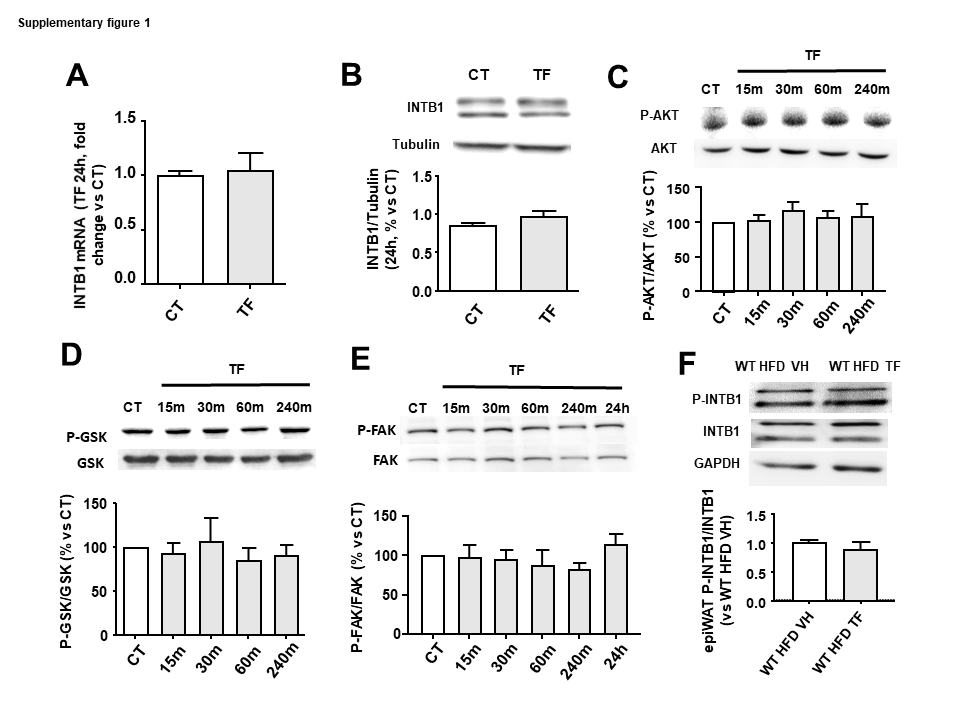


**FIGURE S1: TF does not modify INTB1 and FAK expressions after 24 h, phosphorylation of AKT, GSK and FAK from 15 to 240 minutes, FAK at 24 h or INTB1 after 2 weeks administration in vivo.** A) to E): Deprived differentiated adipocytes from c3H10T1/2 were treated with TF 50 µM or vehicle (CT) for the indicated times. A) 24 h fold changes of INTB1 mRNA expression were analyzed by RT-qPCR and normalized to β-actin B), Representative immunoblots and densitometric analysis of total INTB1 levels after 24 h, normalized to tubulin levels. C) and D) Representative immunoblots and densitometric analysis of AKT phosphorylated at Ser473 (P-AKT) and GSK3β phosphorylated at Wer9 (P-GSK) respectively, between 15 and 240 min and normalized to total AKT or GSK3β protein contents. E) Representative immunoblots and densitometric analysis of FAK phosphorylated at Tyr297 (P-FAK) between 15 min and 24 h and normalized to total FAK protein content. F) Control mice (WT) were challenged to high fat diet (HFD) for 2 weeks and subjected to TF (50 microg/Kg/day, i.p.) or vehicle (VH). Afterward, animals were fasted overnight, weighed, sacrificed and epididymal white AT depots (epiWAT) were dissected and processed. Representative immunoblots and densitometric analysis of phosphorylated INTB1 at Thr788/9 (P-INTB1) vs total INTB1. Data are shown as mean ± SEM. N=6-12.


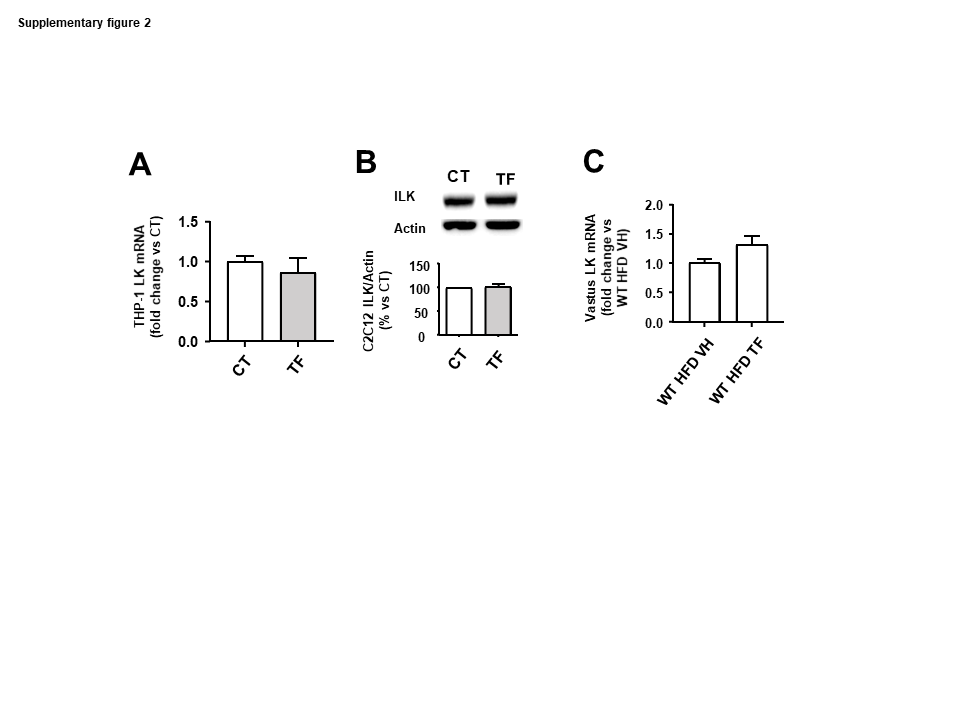


**FIGURE S2: TF does not modify ILK expression in monocytes in vitro or in skeletal muscle in vitro and in vivo.** A) Deprived cultured monocytes THP1 and B) myoblasts C2C12 were treated with TF 50 µM or vehicle (CT) 24 h and ILK mRNA expression fold changes were analyzed by RT-qPCR and normalized to β-actin. C) Control mice (WT) were challenged to high fat diet (HFD) for 2 weeks and subjected to TF (50 microg/Kg/day, i.p.) or vehicle (VH). Afterward, animals were fasted overnight, weighed, sacrificed and vastus lateralis were dissected. Fold changes of ILK mRNA expression analyzed by RT-qPCR and normalized to β-actin. Data are shown as mean ± SEM. N=6-12.
